# Energetic Basis for Exercise-Induced Pulmonary Congestion in Heart Failure With Preserved Ejection Fraction

**DOI:** 10.1161/CIRCULATIONAHA.121.054858

**Published:** 2021-11-08

**Authors:** Matthew K. Burrage, Moritz Hundertmark, Ladislav Valkovič, William D. Watson, Jennifer Rayner, Nikant Sabharwal, Vanessa M. Ferreira, Stefan Neubauer, Jack J. Miller, Oliver J. Rider, Andrew J.M. Lewis

**Affiliations:** University of Oxford Centre for Clinical Magnetic Resonance Research, Radcliffe Department of Medicine (M.K.B., M.H., L.V., W.D.W., J.R., V.M.F., S.N., O.J.R., A.J.M.L.), University of Oxford, UK.; Department of Physics, Clarendon Laboratory (J.J.M.), University of Oxford, UK.; Department of Imaging Methods, Institute of Measurement Science, Slovak Academy of Sciences, Bratislava, Slovakia (L.V.).; Department of Cardiology, Oxford University Hospitals NHS Foundation Trust, John Radcliffe Hospital, UK (J.R., N.S., S.N., O.J.R., A.J.M.L.).; The MR Research Centre and The PET Research Centre, Aarhus University, Denmark (J.J.M.)

**Keywords:** exercise, heart failure, magnetic resonance imaging

## Abstract

Transient pulmonary congestion during exercise is emerging as an important determinant of reduced exercise capacity in heart failure with preserved ejection fraction (HFpEF). We sought to determine whether an abnormal cardiac energetic state underpins this process.

**Methods:**

We recruited patients across the spectrum of diastolic dysfunction and HFpEF (controls, n=11; type 2 diabetes, n=9; HFpEF, n=14; and severe diastolic dysfunction attributable to cardiac amyloidosis, n=9). Cardiac energetics were measured using phosphorus spectroscopy to define the myocardial phosphocreatine to ATP ratio. Cardiac function was assessed by cardiovascular magnetic resonance cine imaging and echocardiography and lung water using magnetic resonance proton density mapping. Studies were performed at rest and during submaximal exercise using a magnetic resonance imaging ergometer.

**Results:**

Paralleling the stepwise decline in diastolic function across the groups (E/e′ ratio; *P*<0.001) was an increase in NT-proBNP (N-terminal pro-brain natriuretic peptide; *P*<0.001) and a reduction in phosphocreatine/ATP ratio (control, 2.15 [2.09, 2.29]; type 2 diabetes, 1.71 [1.61, 1.91]; HFpEF, 1.66 [1.44, 1.89]; cardiac amyloidosis, 1.30 [1.16, 1.53]; *P*<0.001). During 20-W exercise, lower left ventricular diastolic filling rates (r=0.58; *P*<0.001), lower left ventricular diastolic reserve (r=0.55; *P*<0.001), left atrial dilatation (r=–0.52; *P*<0.001), lower right ventricular contractile reserve (right ventricular ejection fraction change, r=0.57; *P*<0.001), and right atrial dilation (r=–0.71; *P*<0.001) were all linked to lower phosphocreatine/ATP ratio. Along with these changes, pulmonary proton density mapping revealed transient pulmonary congestion in patients with HFpEF (+4.4% [0.5, 6.4]; *P*=0.002) and cardiac amyloidosis (+6.4% [3.3, 10.0]; *P*=0.004), which was not seen in healthy controls (–0.1% [–1.9, 2.1]; *P*=0.89) or type 2 diabetes without HFpEF (+0.8% [–1.7, 1.9]; *P*=0.82). The development of exercise-induced pulmonary congestion was associated with lower phosphocreatine/ATP ratio (r=–0.43; *P*=0.004).

**Conclusions:**

A gradient of myocardial energetic deficit exists across the spectrum of HFpEF. Even at low workload, this energetic deficit is related to markedly abnormal exercise responses in all 4 cardiac chambers, which is associated with detectable pulmonary congestion. The findings support an energetic basis for transient pulmonary congestion in HFpEF.

## Abstract 2

Supplemental Digital Content is available in the text.

**Clinical Perspective**

**What Is New?**

- A gradient of myocardial energetic impairment exists across a spectrum of heart failure with preserved ejection fraction phenotypes of increasing clinical severity and worsening diastolic function.
- A greater degree of myocardial energetic deficit is linked to impaired left ventricular systolic and diastolic functional reserve, to altered right ventricular reserve, and to exercise-induced pulmonary congestion assessed using novel pulmonary proton density magnetic resonance imaging.

**What Are the Clinical Implications?**

- A subgroup of patients with heart failure with preserved ejection fraction demonstrate transient pulmonary congestion during exercise, which can be noninvasively assessed using exercise cardiovascular magnetic resonance imaging and pulmonary proton density mapping.
- Manipulating myocardial energy metabolism may be a promising strategy to improve cardiac function and reduce pulmonary congestion in heart failure with preserved ejection fraction.

**Editorial, see p 1679**

Cardinal symptoms of heart failure with preserved ejection fraction (HFpEF) include exertional dyspnea and effort intolerance.^1^ Recent studies implicate abnormal cardiac mitochondrial function and energetics in the central pathogenesis of HFpEF.^2–7^ Whereas metabolic syndromes including type 2 diabetes (T2D) and obesity are clearly linked to an increased risk of developing HFpEF,^8,9^ whether an abnormal cardiac energetic reserve limits the ability of the human heart to respond appropriately to exercise and underlies symptoms in HFpEF is unclear.^10^

Exercise-induced pulmonary congestion has emerged as a common pathway responsible for dyspnea in many patients with HFpEF.^11,12^ Left ventricular (LV) diastolic dysfunction leads to high left-sided filling pressures during exercise, which drives transient pulmonary transcapillary fluid transudation. Furthermore, high systemic venous pressures, in part reflecting abnormal right heart functional reserve, decrease the rate of clearance of this extravasated fluid through pulmonary lymphatics.^11^ Given that the active phase of diastole and ventricular contractile reserve are both susceptible to energetic deficit,^13^ it is plausible that an abnormal cardiac energetic state may underpin these processes.

We hypothesized that not only would a gradient of myocardial energetic impairment exist across the spectrum of HFpEF and diastolic dysfunction, but that this energetic deficit would predispose to LV diastolic dysfunction and right ventricular impairment during exercise. We also hypothesized that these cardiac functional changes during exercise would then predispose to the development of transient pulmonary congestion.

To investigate this, we designed and conducted a basket trial covering the physiologic spectrum of HFpEF severity. We noninvasively assessed cardiac energetics in this cohort using phosphorus magnetic resonance spectroscopy and combined real-time free-breathing volumetric assessment of whole-heart mechanics with a novel pulmonary proton-density magnetic resonance imaging (MRI) sequence to detect lung congestion both at rest and during submaximal exercise.

## Methods

The study received approval from a Research Ethics Committee (South Central Oxford C, 15/SC/0004). Research was performed in accordance with institutional procedures and the Declaration of Helsinki. All participants were recruited from clinical activity at the Oxford University Hospitals National Health Service Foundation Trust or by poster advertisement. All participants provided written informed consent. All data are available on reasonable request from the corresponding author.

### Study Design

**Figure 1.**
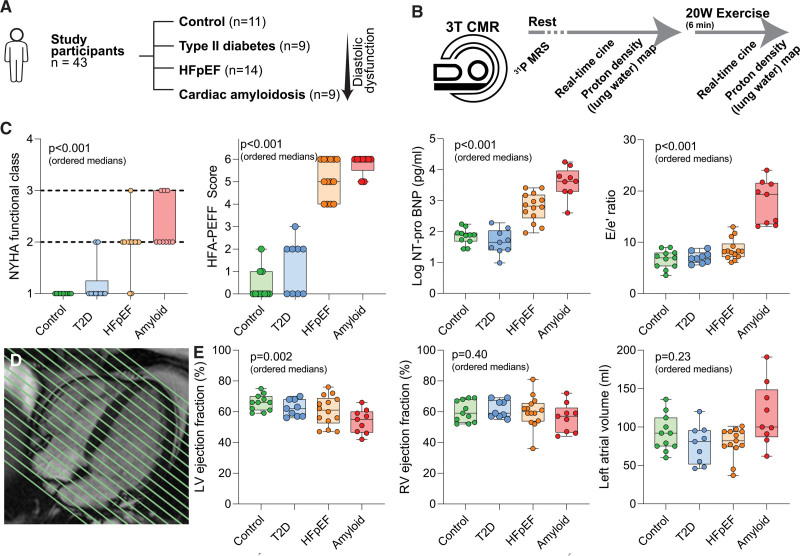
**Baseline clinical and cardiovascular magnetic resonance parameters. A**, Participants and study groups for the basket trial design. **B**, Cardiovascular magnetic resonance (CMR) imaging and spectroscopy protocol at rest and during exercise using a magnetic resonance ergometer. **C**, Baseline clinical characteristics for New York Heart Association (NYHA) functional class, HFA-PEFF score, NT-proBNP (N-terminal pro-brain natriuretic peptide), and E/e′ ratio across the spectrum of diastolic dysfunction. **D**, CMR horizontal long axis image demonstrating whole-heart short axis coverage using real-time imaging. **E**, CMR parameters for left ventricular (LV) ejection fraction, right ventricular (RV) ejection fraction, and left atrial volume across the 4 study groups. *P* values (ordered medians) reported are the result of Jonckheere-Terpstra test across the groups. HFpEF indicates heart failure with preserved ejection fraction; MRS, magnetic resonance spectroscopy; and T2D, type 2 diabetes.

The basket trial design included 43 participants across 4 groups covering the spectrum of diastolic dysfunction and HFpEF, assessed using clinical markers of severity and the HFA-PEFF score,^14^ as follows (Figure 1A):

Group 1: age-matched controls (n=11; participants with no history of significant cardiovascular disease or cardiac symptoms).

Group 2: patients with cardiometabolic risk factors, including T2D (n=9), but without a diagnosis of HFpEF, with HbA1c >6.5%, on oral hypoglycemic agents, HFA-PEFF score ≤3, and no symptoms of heart failure. T2D was selected because it is the disease most convincingly linked to the risk of development of HFpEF^15^; other HFpEF risk factors including higher body weight and hypertension were also common in this group.

Group 3: patients with established HFpEF (n=14): clinical diagnosis of HFpEF from an experienced cardiologist, history of symptomatic breathlessness on exertion, no alternative explanation for effort intolerance, preserved LV ejection fraction (LVEF) >45%, and HFA-PEFF score ≥4, incorporating echocardiographic evidence of diastolic dysfunction and elevated NT-proBNP (N-terminal pro-brain natriuretic peptide) level.

Group 4: severe diastolic dysfunction attributable to cardiac amyloidosis (n=9, of whom 8 had a clinical diagnosis of transthyretin wild-type amyloidosis and 1 had a clinical diagnosis of AL amyloidosis): positive ^99m^Tc-DPD scan (Perugini grade 2 or 3)^16^ or diagnostic appearances on cardiovascular magnetic resonance (CMR) imaging, evidence of raised filling pressures or restrictive filling pattern on echocardiography, and HFA-PEFF score ≥5. A cardiac amyloidosis group was chosen to provide positive control data for the experiment, representing the most severe form of diastolic dysfunction.

Participants were excluded if they had known primary lung disease or pulmonary hypertension, obstructive coronary artery disease, more than moderate valvular heart disease, uncontrolled hypertension, arrhythmia with an uncontrolled ventricular response >110 bpm,^17^ inability to exercise, or any other contraindication to magnetic resonance examination.

### Phosphorus Magnetic Resonance Spectroscopy to Assess Myocardial Energetics

Phosphorus magnetic resonance spectroscopy was performed at rest on a 3T MRI scanner (Magnetom Trio; Siemens Healthineers; Figure 1B). Participants were positioned prone over the center of a 3-element dual-tuned ^1^H/^31^P surface coil in the isocenter of the MRI system. A nongated 3D acquisition-weighted ultrashort echo time (UTE) chemical shift imaging sequence was used with saturation bands placed over liver and skeletal muscle, as previously described.^18^ All spectra were analyzed using a semiautomated fitting of data from within OXSA toolbox^19^ (ie, a MATLAB implementation of the AMARES fitting routine by an experienced operator [M.K.B., who has 3 years of CMR experience]). The fitted phosphocreatine (PCr) and ATP signals were corrected for partial saturation, using literature values,^20^ before calculating the PCr/ATP ratio as PCr/average ATP or PCr/γ-ATP depending on spectral quality. The reported PCr/ATP was averaged over 2 basal septal voxels and corrected for blood signal contamination.^21^ Further details are provided in the Supplemental Material.

### Cardiovascular MRI at Rest and During Exercise Stress

Cardiovascular MRI was also performed on a 3T MRI scanner (Magnetom Prisma; Siemens Healthineers) using a 32-channel phased array coil (Figure 1B). After standard planning, a retrospectively gated, highly accelerated, compressed sensing, free-breathing cine imaging sequence was used to acquire a short axis stack covering the entire heart, including both ventricles and atria, within 60 seconds. Imaging parameters are provided in the Supplemental Material. T1 mapping was performed using the ShMOLLI method (shortened modified look locker inversion recovery), as previously described.^22^ Lung imaging was performed using a custom proton density–weighted sequence in a standardized axial slice at the level of the pulmonary arteries.

Exercise stress was then performed using a CMR-compatible pedaling ergometer in the supine position (Cardio Step; Ergospect GmbH). The exercise protocol comprised a fixed workload of 20 W for 6 minutes. This protocol was selected to reflect the measures commonly used for invasive hemodynamic studies in HFpEF.^11^ Repeat whole-heart cine images were acquired during the final minute of the exercise period; repeat lung imaging was performed immediately on cessation of exercise.

### Development of Lung Water Sequence

To rapidly and robustly quantify dynamic changes in lung water, a custom proton density lung mapping sequence was developed. This used a constant low flip angle, multiecho UTE pulse sequence with a golden-angle radial-out sampling scheme, and out-of-slice and fat saturation schemes (Figure S1).^23^ The sequence was first validated using Bloch equation simulations and analytic knowledge of the gradient echo signal equation (Figures S2 and S4). With the low flip angle used (5°) and the ability of UTE sequences to adequately resolve lung parenchyma, scan parameters were chosen to ensure an approximately linear response of signal intensity to proton density within the lung in its physiologic range. A novel modified radial post hoc reconstruction algorithm was developed to ensure that residual exercise-induced motion artefacts were aliased outside the lung field of view (Figure S3). This resulted in a compact sequence that was able to quantify a linear increase in water density within the pores of a ≈100 µm^3^ sponge phantom designed as a mimetic of lung parenchyma. Further details are provided in the Supplemental Material.

### Image Analysis

Image analysis for atrial and ventricular volumes was performed offline using a semiautomated system (cmr42 version 5.10.1; Circle Cardiovascular Imaging Inc.) in accordance with Society for Cardiovascular Magnetic Resonance guidelines.^24^ Endocardial and epicardial contours were curated manually for all cardiac chambers at rest and stress by an independent operator (M.K.B.; 3 years CMR experience) and checked sequentially by 2 further independent operators (A.J.M.L.; 10 years CMR experience and O.J.R.; 16 years CMR experience). Diastolic volume–time curves were generated by contouring short axis stacks at rest and stress in each slice and phase over 1 cardiac cycle. Right ventricular (RV) to pulmonary artery (PA) coupling was assessed using downstream physiologic surrogates of right heart reserve (RV ejection fraction augmentation and right atrial [RA] dilation during exercise) as well as the RV stroke volume (SV) to end-systolic volume (ESV) ratio as previously described.^25,26^

Lung water quantitation was performed by contouring the lung peripheries in matched axial slices at rest and stress by a single operator (O.J.R.) using ImageJ software.^27^ Repeat contouring was performed in a blinded fashion to assess intra- and interoperator and test-retest variability.

### Echocardiography

Echocardiography was performed on a GE Vivid I system using a standardized protocol including parasternal long and short axis views as well as apical 2, 3, and 4-chamber views for chamber quantification. Color Doppler assessments were performed to exclude significant valvular heart disease. Pulsed-wave Doppler assessment of mitral valve inflow was used to calculate E/A ratio. Tissue Doppler velocities were measured at the medial and lateral mitral valve annulus to determine E/e′ ratios. Continuous wave Doppler was used to assess tricuspid regurgitation velocity for estimation of systolic pulmonary artery pressure (sPAP).

### Statistical Analysis

Data are presented as median (interquartile range) or number (%). Ordered medians were compared using the Jonckheere-Terpstra test, according to the study design along the spectrum of diastolic dysfunction: controls > T2D > HFpEF > cardiac amyloid. Between-group differences were compared using Wilcoxon signed-rank test, Kruskal-Wallis test with Dunn post hoc comparisons, or Fisher exact test as appropriate. Pearson correlation (r) and linear regression were used. Repeat analyses were performed with exclusion of the amyloid patient group. To explore the potential for an indirect effect of myocardial energetics on lung water transudation through either diastolic function or right ventricular function, mediator multiple regression was performed according to the method of Preacher and Hayes,^28^ with 5000 sample bootstrapping of indirect effects (dependent variable, lung water transudation; independent variable, PCr/ATP; moderators, E/e′, RVEF change during exercise, and RA volume change during exercise). A 2-tailed probability value of *P*<0.05 was considered significant. Analyses were performed using R Studio (RStudio Team [2020]. RStudio: Integrated Development for R. RStudio, PBC, Boston, MA) and GraphPad Prism (version 9.0.2 for Windows; GraphPad Software, San Diego, CA).

## Results

### Baseline Characteristics

Baseline characteristics for the study participants are presented in Table 1. When the study groups are ordered control > T2D > HFpEF > amyloid, there was a stepwise increase in the severity of conventional HFpEF markers (Figure 1C), including symptom burden (New York Heart Association status; *P*<0.001), HFA-PEFF score (from 0 [0, 1] to 6 [5.5, 6]; *P*<0.001), serum NT-proBNP level (from 78 [49, 91] to 4217 pg/mL [1964, 9978]; *P*<0.001), diastolic dysfunction (E/e′ ratio from 6.9 [5.4, 8.0] to 19.3 [13.5, 21.6]; *P*<0.001), and estimated sPAPs (sPAP from 9.1 [7.8, 10.8] to 40.7 mm Hg [22.5, 53.5]; *P*<0.001). Native T1 times were also increased (from 1143 [1116, 1163] to 1346 [1278, 1349]; *P*<0.001). These results all remained strongly significant even with exclusion of the amyloid group (Table S1). Participants in groups 1 to 3 were well-matched for age (*P*=NS). Participants in the HFpEF group were also matched to the T2D group for body mass index and blood pressure (*P*=NS for all comparisons).

**Table 1.**
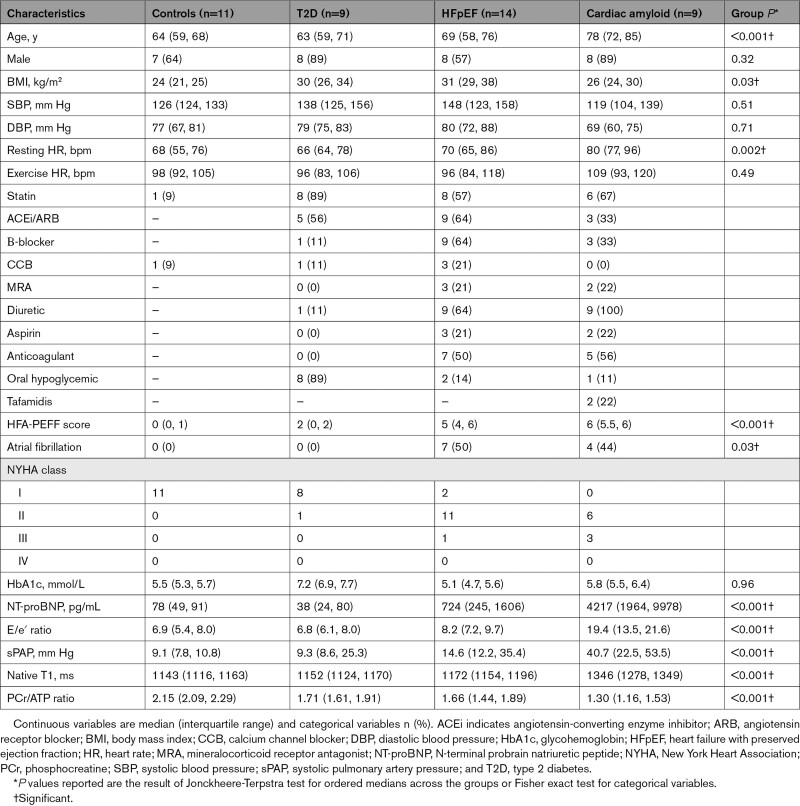
Baseline Characteristics

### Left and Right Ventricular Structure and Function at Rest

Although there were no significant differences in CMR-derived LV and RV end-diastolic volumes (EDVs) at rest (Table 2), there was a stepwise increase in median total LV mass index across the ordered groups (57 g/m^2^ [49, 64] versus 104 g/m^2^ [81, 122]; *P*<0.001). Although all patients had an LVEF >45% by echocardiography at the time of study entry, patients with cardiac amyloidosis had a significantly lower LVEF than age-matched controls assessed using CMR (55% [47, 60] versus 66% [61, 70]; *P*=0.002; Figure 1D and 1E).

**Table 2.**
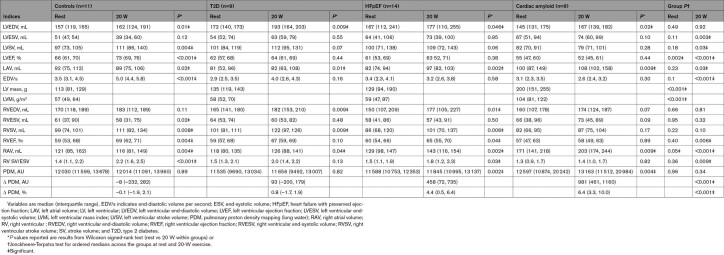
Cardiac and Pulmonary Indices at Rest and During 20-W Exercise

### Myocardial Energetics Across the Spectrum of Diastolic Dysfunction and HFpEF

**Figure 2.**
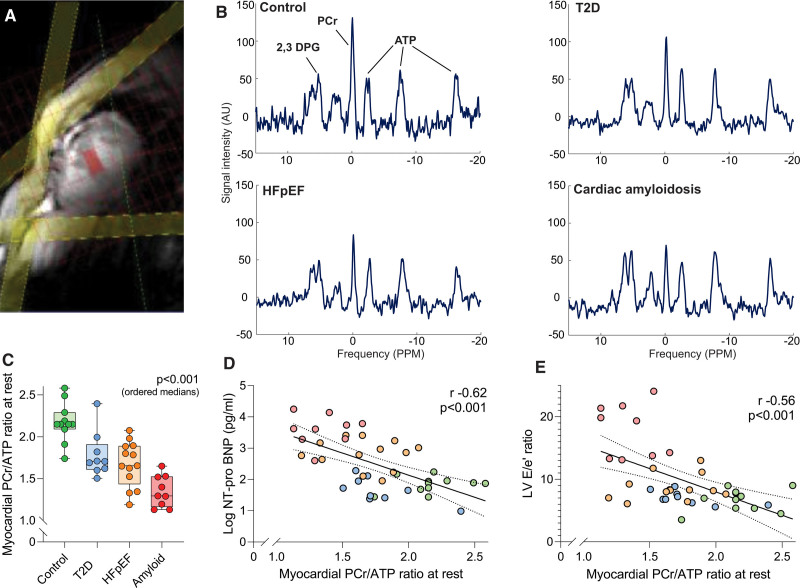
**Cardiac energetics across the spectrum of diastolic dysfunction. A**, Phosphorus magnetic resonance spectroscopy acquisition strategy with a voxel placed in the midventricular septum (red) and saturation bands (yellow) placed over skeletal muscle and the liver. **B**, Representative cardiac ^31^P spectra for the different study groups showing (**C**) progressive depletion of phosphocreatine (PCr)/ATP and associations with (**D**) NT-proBNP (N-terminal pro-brain natriuretic peptide) and (**E**) left ventricular (LV) E/e′ ratio. *P* values (ordered medians) reported are the result of Jonckheere-Terpstra test across the groups or from linear regression analysis. DPG indicates diphosphoglycerate; and T2D, type 2 diabetes.

Cardiac energetic assessment using phosphorus magnetic resonance spectroscopy demonstrated a stepwise decrease in median myocardial PCr/ATP across the ordered groups (control 2.15 [2.09, 2.29], T2D 1.71 [1.61, 1.91], HFpEF 1.66 [1.44, 1.89], and cardiac amyloidosis 1.30 [1.16, 1.53]; *P*<0.001; Figure 2A–2C). There was a 23% median decrease in PCr/ATP in HFpEF and 40% median decrease in PCr/ATP in cardiac amyloidosis compared with controls. This progressive energetic impairment across the groups remained strongly significant even with exclusion of the amyloid group (Table S1). The degree of energetic deficit correlated with conventional biomarkers of HFpEF severity, including log NT-proBNP (r=–0.62; *P*<0.001; Figure 2D) and echocardiographic E/e′ ratio (r=–0.56; *P*<0.001; Figure 2E). A lower PCr/ATP ratio was linked to a higher body mass index (r=–0.35; *P*=0.02) but not to HbA1C level (r=–0.12; *P*=0.47). A lower PCr/ATP ratio was also linked to higher native T1 times across the ordered groups (r=–0.59; *P*<0.001). When participants were ordered by New York Heart Association functional class (I > II > III > IV), there was a progressive decline in myocardial PCr/ATP (*P*=0.03).

These findings are consistent with a gradient of myocardial energetic deficit across the HFpEF spectrum, which parallels the clinical and conventional biomarkers of disease severity.

### Abnormal LV and RV Systolic Responses to Exercise in HFpEF

All participants successfully completed the submaximal magnetic resonance exercise protocol of 6 minutes of fixed 20-W supine ergometry or achieved volitional exhaustion. Real-time, free-breathing CMR volumes and functional indices acquired at rest and during 20-W exercise stress are reported in Table 2.

**Figure 3.**
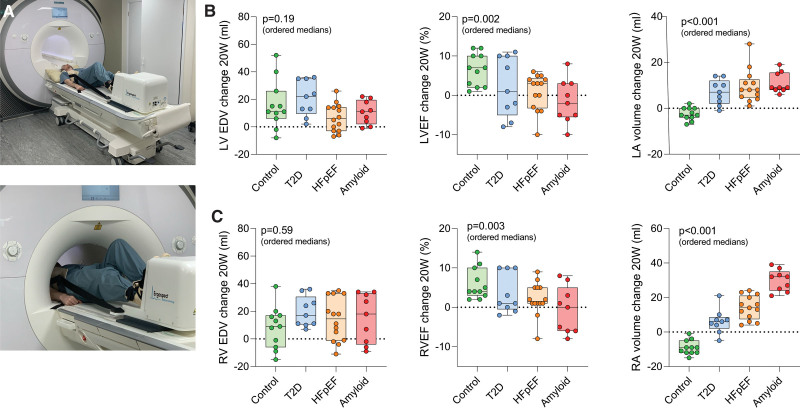
**Exercise cardiovascular magnetic resonance parameters. A**, Cardiovascular magnetic resonance (CMR) compatible exercise ergometer. **B**, Change in CMR parameters between rest and stress for left ventricular end-diastolic volume (LVEDV), left ventricular ejection fraction (LVEF), and left atrial (LA) volume. **C**, Change in CMR parameters between rest and stress for right ventricular end-diastolic volume (RVEDV), right ventricular ejection fraction (RVEF), and right atrial (RA) volume. *P* values (ordered medians) reported are the result of Jonckheere-Terpstra test across the groups. T2D indicates type 2 diabetes.

During exercise, controls demonstrated appropriate augmentation of LV SV (*P*=0.004), LVEF (*P*<0.001), RV SV (*P*=0.008), and RVEF (*P*=0.004). In contrast, across the patient groups (ie, groups 2–4), the exercise inducible change in both LVEF and RVEF was progressively blunted (ΔLVEF *P*=0.002, ΔRVEF *P*=0.003; Figure 3B and 3C). Strikingly, patients with cardiac amyloidosis demonstrated no augmentation in LV SV, LVEF, RV SV, or RVEF (*P*>0.05 for all). Overall, this shows that even at low workload, the normal augmentation of LV and RV systolic function is blunted in diastolic dysfunction and HFpEF and becomes absent in the severest phenotypes of the syndrome.

### Abnormal LV Diastolic Responses to Exercise

**Figure 4.**
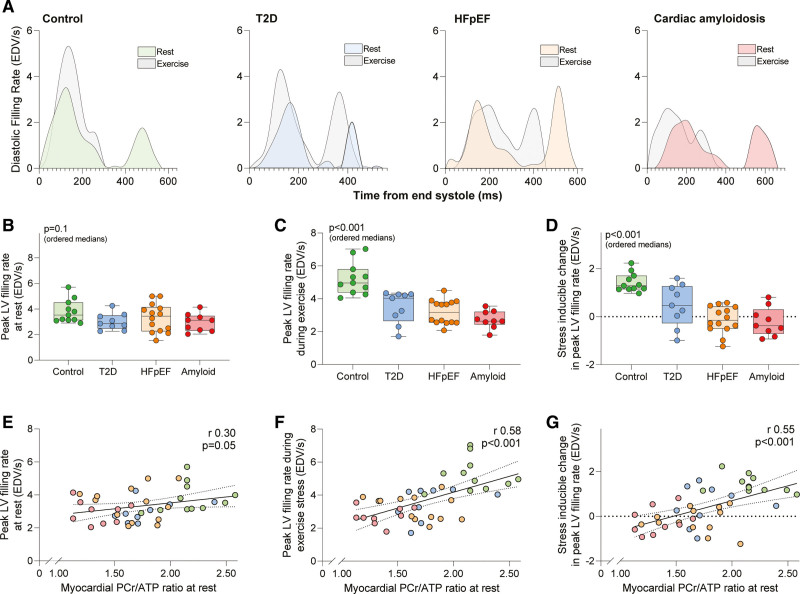
**Cardiac energetics and diastolic filling at rest and during exercise. A**, Representative examples of volume–time curves for the study groups. Second row: (**B**) peak left ventricular (LV) diastolic filling rates at rest, (**C**) peak LV diastolic filling rates during exercise, and (**D**) the stress-inducible change in peak LV diastolic filling rates across the groups. Third row: relationship between cardiac energetics at rest and (**E**) peak LV diastolic filling rates at rest, (**F**) peak LV diastolic filling rates during exercise, and (**G**) the stress-inducible change in peak LV diastolic filling rates across the groups. *P* values (ordered medians) reported are the result of Jonckheere-Terpstra test across the groups or from linear regression analysis. EDV/s indicates end-diastolic volume (in milliliters) per second; HFpEF, heart failure with preserved ejection fraction; PCr, phosphocreatine; and T2D, type 2 diabetes.

Diastolic volume–time curves were plotted from the CMR data to further interrogate cardiac mechanics at rest and during stress; representative examples of these diastolic filling curves are presented in Figure 4A. Peak diastolic filling rates at rest were similar across the groups (*P*=0.1; Figure 4B). Whereas healthy controls appropriately augmented peak diastolic filling during stress (from 3.5 mL/s [3.1, 4.5] to 5.0 mL/s [4.4, 5.8]; *P*<0.001), this was progressively attenuated across the ordered patient groups (*P*<0.001; Figure 4C). The degree of stress-inducible change in peak diastolic filling rates (ie, diastolic reserve) was also progressively blunted and even reversed across the ordered groups (*P*<0.001; Figure 4D). Furthermore, in contrast to the reduction in left atrial (LA) volumes seen during exercise in healthy controls, the patient groups demonstrated stepwise LA dilation during exercise (Figure 3B; *P*<0.001). The degree of LA dilation was associated with diastolic reserve during exercise (r=–0.48; *P*=0.002).

Overall, this shows that the normal increase in LV diastolic filling rates that occurs during exercise in healthy hearts is lower in those with risk factors for HFpEF, absent in HFpEF, and even reversed with lower filling rates during exercise in the most severe forms of HFpEF and diastolic dysfunction. In addition, the resulting LA dilation during exercise, in all but the normal heart, is likely to reflect higher LV filling and LA pressures.

### RV–PA Coupling During Exercise

RV–PA uncoupling was assessed by deriving the RV SV/ESV ratio^25,26^ as well as downstream surrogates such as augmentation of RVEF and RA dilatation during exercise. Estimated sPAP (by echocardiography) at rest was associated with RA dilatation during exercise (r=0.58; *P*<0.001). Across the ordered groups during exercise, there was progressive RV–PA uncoupling (RV SV/ESV; *P*=0.009), failure of RVEF augmentation (*P*=0.003), and significant RA dilatation (*P*<0.001; Figure 3C). The degree of RVEF augmentation (r=0.38; *P*=0.01) and RA dilatation (r=–0.68; *P*<0.001) correlated with LV diastolic reserve during exercise. This shows that normal RV–PA coupling is lost in HFpEF during exercise, and is related to RA dilation, which itself likely reflects higher systemic venous pressure.

### Myocardial Energetics and the Cardiac Response to Exercise

We also investigated links between myocardial energetics and cardiac exercise responses (Figure 4E–4G). During 20-W exercise, lower peak LV diastolic filling rates (r=0.58; *P*<0.001; Figure 4F), LV diastolic reserve (r=0.55; *P*<0.001; Figure 4G), LA dilatation (r=–0.52; *P*<0.001), lower RV contractile reserve (ΔRVEF change, r=0.57; *P*<0.001), and right atrial dilatation (r=–0.71; *P*<0.001) were all linked to lower PCr/ATP across the ordered groups. This highlights that an abnormal resting myocardial energetic state is related to impaired exercise responses of all 4 cardiac chambers.

### Using Pulmonary Proton Density Imaging to Detect Lung Congestion

To detect pulmonary congestion, a novel MRI sequence was designed to provide lung images with signal that would scale linearly with proton density (reflecting water content in this context) across a physiologic range. The sequence was first validated with Bloch-equation simulations (Figure S2), followed by a water-doped sponge phantom validation, establishing that UTE lung water signal was linear within a physiologic range (r=0.98; *P*<0.0001; Figure S5). A linear look-up table was applied to lung images for visualization of pulmonary proton density signal (Figure S6). Limits of agreement on Bland-Altman plots for lung water analysis were highly consistent (Figure S7–S10).

### Exercise-Induced Pulmonary Congestion

**Figure 5.**
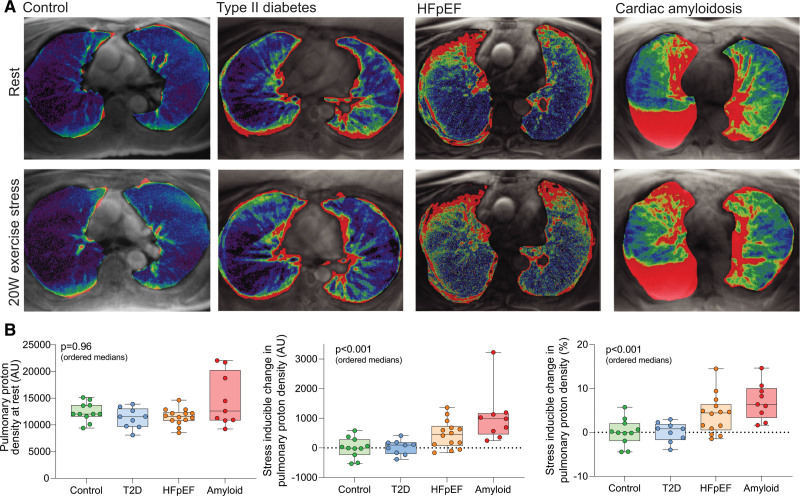
**Pulmonary congestion across the spectrum of diastolic dysfunction. A**, Representative examples of lung water color maps for the study groups at rest and during low-intensity exercise stress. Look-up table details are provided in Figure S6. **B**, Resting pulmonary proton density (lung water) signal for the study groups along with absolute and percentage changes in pulmonary proton density during low-intensity exercise. The participant shown with cardiac amyloidosis has a right-sided pleural effusion. Pleural effusions (n=5) were seen only in the cardiac amyloidosis group and were pre hoc included in the region of interest. However, the magnitude and statistical significance of the stress-inducible change in lung water signal were essentially unchanged on a subsequent retrospective analysis in which pleural effusions were excluded from the region of interest. *P* values (ordered medians) reported are the result of Jonckheere-Terpstra test across the groups. HFpEF indicates heart failure with preserved ejection fraction; and T2D, type 2 diabetes.

Representative pulmonary proton density images acquired during rest and immediately after exercise are shown in Figure 5A. There was no significant difference in rest pulmonary proton density mapping signal across the groups on ordered medians (*P*=0.96; Table 2 and Figure 5B).

Immediately after 20-W exercise, patients with HFpEF and cardiac amyloidosis had a significant increase in both absolute and percentage change in pulmonary proton signal (HFpEF absolute change, 458 AU [72, 735], +4.4% [0.5, 6.4]; *P*=0.002; amyloid absolute change, 981 AU [461, 1160], +6.4% [3.3, 10.0]; *P*=0.004). In contrast, there was no significant change in healthy controls (absolute change, –8 AU [–232, 282], –0.1% [–1.9, 2.1]; *P*=0.89) or patients with T2D (absolute change, 93 AU [–200, 179], +0.8% [–1.7, 1.9]; *P*=0.82). There was a progressive increase in both absolute and percentage change in lung water signal during 20-W exercise across the ordered groups (*P*<0.001 for both; Table 2 and Figure 5B), which remained strongly significant even with exclusion of the amyloid group (*P*=0.005 and *P*=0.009, respectively; Table S2).

**Figure 6.**
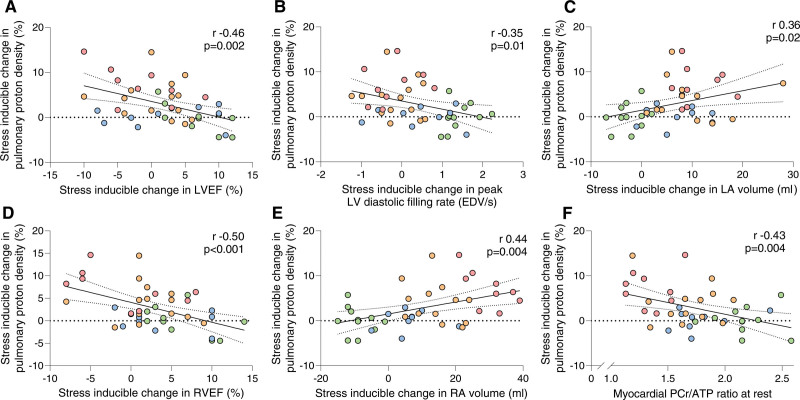
**Cardiac functional reserve, myocardial energetics, and exercise-induced pulmonary congestion.** The relationships between relative change in pulmonary proton signal with exercise and (**A**) stress-inducible change in left ventricular ejection fraction (LVEF), (**B**) stress-inducible change in peak left ventricular (LV) diastolic filling, (**C**) stress-inducible change in left atrial (LA) volume, (**D**) stress-inducible change in right ventricular ejection fraction (RVEF), (**E**) stress-inducible change in right atrial (RA) volume, and (**F**) the underlying energetic basis. *P* values reported are from linear regression analysis. EDV/s indicates end-diastolic volume (in milliliters) per second; and PCr, phosphocreatine.

Lung water signal change during exercise was related to log NT-proBNP (r=0.54; *P*<0.001), resting LV E/e′ (r=0.35; *P*=0.02), and resting native myocardial T1 (r=0.57; *P*<0.001). There was no significant association between echocardiography-estimated sPAP at rest and lung water transudation (r=0.22; *P*=0.15). The stress-inducible change in LVEF (r=–0.46; *P*=0.002; Figure 6A), peak diastolic filling rate (r=–0.33; *P*=0.03), and diastolic reserve (r=–0.35; *P*=0.01; Figure 6B) during exercise were significantly associated with lung water transudation, as was the change in LA volume (r=0.36; *P*=0.02; Figure 6C). Lung water transudation was also significantly associated with markers of RV–PA uncoupling on the basis of RV SV/ESV (r=–0.32; *P*=0.04), RVEF augmentation (r=–0.50; *P*<0.001; Figure 6D), and change in RA volume during 20-W exercise (r=0.44; *P*=0.004; Figure 6E). There was no correlation with RV SV/ESV at rest (r=–0.14; *P*=0.37), highlighting that the relationship between RV–PA uncoupling and lung water transudation is unmasked by exercise. There was a significant association between myocardial PCr/ATP at rest and lung water transudation during stress (r=–0.43; *P*=0.004; Figure 6F). The relationship between PCr/ATP and lung water transudation remained strongly significant after controlling for body mass index (*P*=0.005).

### Assessing the Indirect Effects of Energetics on Lung Water Transudation

On linear regression, although PCr/ATP is related to lung water transudation (β-689; *P*=0.01), a direct effect is not biologically plausible. To assess the indirect effects of myocardial energetics on lung water transudation, moderated multiple regression was performed (dependent variable lung water transudation [absolute change], independent variable PCr/ATP, moderators RVEF change during exercise, RA volume change during exercise, and E/e′). In these models, mediation analysis was tested using 5000 bootstrap resamples to generate a 95% CI (bias corrected) of the indirect effect. This showed that the effects of PCr/ATP are mediated through diastolic function (β-279, CI –811.0 to –5.5), change in RVEF (β-394, CI –987.0 to –33.0), and RA volume change during exercise (β-395, CI –946.5 to –16.5).

Overall, these results highlight that exercise-induced pulmonary congestion is detectable in clinically defined HFpEF groups using MRI proton density mapping. In addition, they show that the degree of pulmonary congestion is not only related to the degree of impairment of the cardiac response to exercise, but also to HFpEF severity and the extent of underlying myocardial energetic deficit.

## Discussion

We investigated the links among cardiac energetics, myocardial functional reserve, and pulmonary congestion across the spectrum of HFpEF and diastolic dysfunction. We demonstrated a graded cardiac energetic deficit that correlated to conventional markers of disease severity including New York Heart Association class, NT-proBNP level, and echocardiographic indices of diastolic function. This energetic deficit was linked to impaired myocardial functional reserve and RV–PA uncoupling during low-intensity exercise and to greater pulmonary congestion during exercise.

### Myocardial Energetics in HFpEF

Although there are numerous etiologic risk factors for HFpEF, including aging, T2D, obesity, and hypertension, all are linked to a myocardial energetic deficit.^15,29–36^ Electron micrographs of LV myocardium demonstrate mitochondrial disarray and fragmentation in HFpEF,^4^ transcriptomic studies show downregulation of key mitochondrial pathways,^5^ and novel mouse models highlight links between metabolic stress^6^ and matrix expansion, which can be targeted using metabolic modulators.^7^ While the mechanisms of myocardial energetic deficit may vary according to the precise etiology, this energetic impairment may be a unifying feature of HFpEF. Active myocardial relaxation through SERCA (sarcoplasmic reticulum Ca^2+^ ATPase) is a highly energy-dependent process because, although the myosin ATPase consumes a greater quantity of ATP than SERCA, SERCA function requires the highest chemical driving force of all cardiac ATPases (ΔG_≈ATP_ ≈–53 kJ.mol^–^^1^).^37^ Diastole thus has the greatest susceptibility to energetic deficit. In line with this, the decreasing PCr/ATP seen here across the groups confirms that a gradient of myocardial energetic deficit extends across the spectrum of progressive diastolic impairment. This builds on an extensive body of preclinical and early human mechanistic data demonstrating an energetic deficit in HFpEF and confirms that these findings have relevance to the human phenotype.^38^

### Myocardial Energetics in Cardiac Amyloidosis

It is not surprising that energetic deficit exists in cardiac amyloidosis because extracellular protein deposition is highly likely to increase the energetic cost of contraction and relaxation attributable to increased tissue stiffness. However, the degree of energetic deficit in this group in this study is among the most severe reported in any cardiovascular disease, and the mechanisms warrant further study. Recent data highlight major microvascular dysfunction and oxygen starvation in amyloidosis, which would be expected to cause energetic deficit.^39^ Abnormal protein deposition might also compromise the normal cycling of redundant mitochondrial fragments across cell membranes,^40^ whereas circulating prefibrillar proteins may directly cause toxicity and increase oxidative stress.^41^

### Left Heart Response to Exercise

Although patients in this study had a preserved LVEF at rest, there was a markedly abnormal left heart response to low-intensity exercise, which was characterized by lower peak LV diastolic filling rates, blunted LV contractile reserve, and LA dilatation. We observed clear links between impaired cardiac energetics and this abnormal response to exercise. Lower cardiac energetics were related to lower peak LV filling rates, lower LV diastolic reserve, and LA dilatation during exercise, consistent with higher LA pressure in those with an underlying energetic deficit. It follows that if LA pressure is higher, then pulmonary capillary hydrostatic pressure is also higher, predisposing to pulmonary congestion through Starling forces and thus impairing pulmonary gas exchange.^11,12,42–44^

The association between exercise-induced high left heart filling pressures and symptoms is well described in HFpEF,^12^ but is less clear in heart failure with reduced ejection fraction.^45–47^ We speculate that the key hemodynamic differences between HFpEF and heart failure with reduced ejection fraction include a more profound impairment of cardiac output reserve, which may result in tissue or skeletal muscle hypoperfusion, resulting in effort intolerance and activation of central feedback pathways, resulting in dyspnea. We also speculate that the right heart and systemic venous responses to exercise may be more profoundly dysfunctional in HFpEF than heart failure with reduced ejection fraction, resulting in and compounding greater fluid transudation for a given pulmonary pressure attributable to inadequate lymphatic clearance.

### Right Heart Response to Exercise

The degree of myocardial energetic deficit was also clearly linked to abnormal right heart exercise responses, including failure of RVEF augmentation and striking RA dilatation. These findings would be in keeping with elevated systemic venous pressure caused by stagnation, which would be expected to exacerbate pulmonary congestion by reducing lung water clearance through pulmonary lymphatics to the central veins.^11,48^ Consistent with this, RA dilatation during exercise was also strongly linked to lung water transudation. The combination of increased pulmonary capillary hydrostatic pressure and reduced lymphatic drainage has also been shown to further worsen pulmonary congestion during exercise in animal models.^48^

Given that a lower myocardial PCr/ATP ratio in this study was related to these abnormalities in both left and right heart exercise responses, it is unsurprising that energetic deficit was linked to exercise-induced pulmonary congestion. These findings suggest a pathway by which impaired energetics are linked to patient symptoms by limiting cardiac functional reserve during stress and thereby promoting pulmonary congestion. As such, targeting myocardial energetics may be a rational strategy to improve cardiac function and reduce pulmonary congestion in HFpEF.

### Pulmonary Congestion as a Distinct Phenotype of HFpEF

The significant heterogeneity of clinical HFpEF syndromes is a major challenge to efforts to develop new therapies to improve symptoms and prognosis. The imaging assessments presented may have future value for defining new endotypes and mechanisms within HFpEF; for example, by identifying patients in whom dyspnea most clearly relates to pulmonary congestion during exercise.^11,43,49–51^ As such, pulmonary proton density mapping might well evolve to become a noninvasive alternative to right heart catheterization for the diagnosis of HFpEF.

### Limitations

One theoretical limitation of the pulmonary proton density imaging performed in this study is the inability to distinguish intravascular from extravascular lung water; however, the absence of a significant change in lung signal in the non-HFpEF groups provides confidence that factors such as greater pulmonary perfusion during exercise are not responsible for the signal change. Invasive hemodynamic assessment is not routinely performed in our health care system, although the hemodynamic response to exercise in HFpEF has been well characterized previously and patients with HFpEF and cardiac amyloidosis met stringent clinical criteria. Although blood pressure measurements were not obtained during exercise, this is not a major confounder given the low-intensity nature of the exercise protocol. We did not administer gadolinium to assess extracellular volume fraction because of the potentially confounding effect of exercise on the microcirculation.

### Conclusions

A gradient of myocardial energetic deficit exists across the spectrum of HFpEF and is associated with abnormal cardiac exercise responses and exercise-induced pulmonary congestion. These findings identify an energetic basis for transient pulmonary congestion in HFpEF.

## Acknowledgments

The authors thank Adrienne G. Siu, Iulius Dragonu, Luca Biasiolli, and Matthew D. Robson for providing the original ultrashort echo time sequence on which the lung water sequence was developed.

## Sources of Funding

This study was principally funded by a British Heart Foundation Intermediate Clinical Research Fellowship (FS/16/70/32157 to Dr Rider). Dr Burrage acknowledges support from a British Heart Foundation Clinical Research Training Fellowship (FS/19/65/34692). Dr Valkovič is supported by the Sir Henry Dale Fellowship, jointly funded by the Royal Society and the Wellcome Trust (221805/Z/20/Z), and acknowledges support of the Slovak Grant Agencies VEGA (2/0003/20) and APVV (19–0032). Dr Miller was supported by a Novo Nordisk Postdoctoral Fellowship run in conjunction with the University of Oxford. S. Neubauer, A.J.M. Lewis, and O.J. Rider acknowledge support from the British Heart Foundation Oxford Center of Research Excellence. A.J.M. Lewis, M.K. Burrage, and S. Neubauer acknowledge support from the National Institute for Health Research Oxford Biomedical Research Center. A.J.M. Lewis acknowledges funding from the Academy of Medical Sciences (SGL021\1038).

## Disclosures

The views expressed are those of the authors and not necessarily those of the UK National Health Service, the UK National Institute of Health Research, or the UK Department of Health and Social Care.

## Supplemental Material

Methods and Results

Figures S1-S10

Tables S1 and S2

References 52–62

## Supplementary Material


