# Supplementary material for: Energetic Basis for Exercise-Induced Pulmonary Congestion in Heart Failure With Preserved Ejection Fraction
Source: Circulation. 2021 Nov 8;144(21):1664–78. doi: 10.1161/CIRCULATIONAHA.121.054858 (PMC8601674; doi:10.1161/CIRCULATIONAHA.121.054858)
Supplement: Supplementary file 1 [file cir-144-1664-s001.pdf]

Burrage et al.: Supplemental Material

Supplemental Methods and Results

1 Blood sampling

Participants underwent blood sampling prior to exercise. Assessments, provided by the standard accredited hospital laboratory, included a full blood count, serum creatinine and estimated glomerular filtration rate (eGFR), HbA1c, and the N-terminal prohormone of brain natriuretic peptide (NT-proBNP).

2 ^{31}P MR Spectroscopy to assess myocardial energetics

^{31}P MR spectroscopy was performed on a 3 Tesla Magnetom Tim Trio MRI scanner (Siemens Healthineers, Erlangen, Germany). Participants were positioned prone over the centre of a 3-element dual-tuned ^{1}H/^{31}P surface coil in the isocenter of the MR system. A non-gated 3D acquisition-weighted ultra-short echo time chemical shift imaging sequence was used with saturation bands placed over liver and skeletal muscle as previously described.[18] The acquisition matrix contained $16 \times 8 \times 8$ voxels with field of view of $240 \times 240 \times 200\,\mathrm{mm}^3$, i.e. nominal voxel size was $11.25\,\mathrm{mL}$. The centre frequency for both excitation and reception was set $250\,\mathrm{Hz}$ lower than the resonant frequency of phosphocreatine (PCr) to ensure the uniform excitation of the whole ^{31}P spectrum equally. To increase the achievable SNR, the nuclear Overhouser effect (NOE) was exploited via the addition of appropriate RF pulses into the UTE-CSI sequence. The TR was adjusted (ranging from 900–1100 ms) to minimize scan time and stay within the specific absorption rate (SAR) limits for each patient. The exact excitation flip angle used was calculated retrospectively based on using both Biot Savart's Law, the known coil geometry, and the relative position of the coil and coil loading through a series of specific localisers, which were determined from a series of inversion recovery acquisitions of a phantom placed inside the coil housing.[18]

Spectral analysis and reconstruction was performed using the OXSA Toolkit with explicit calculation of both flip angle and peak partial saturation, correcting for the differing T_1 of each metabolite given a particular TR, prior to fitting the phosphorus spectrum via AMARES, as described in detail elsewhere.[19]

3 Free-breathing cine imaging

A retrospectively-gated, four-fold accelerated, compressed sensing, free-breathing 2D cine imaging sequence was used to acquire a short axis stack covering the entire heart, including both ventricles and atria. This BEAT-2CV sequence was based on a modified bSSFP sequence, with a variable-density trajectory, and reconstructed using the open-source toolkit Gadgetron, as described previously.[53,54,55]

Exact sequence parameters were optimised on a per-patient basis, but these were typically a TR of 42–43 ms, determined to be minimal but subject to dynamically evolving SAR and FOV constraints; TE 1.17 ms, determined by a desire for a fixed readout bandwidth of 1302 Hz/Px, flip angle 30–40°, slice thickness 8 mm, FOV typically $340 \times 340\,\mathrm{mm}^2$ (but increased on a per-patient basis to avoid spatial aliasing). The base resolution was 160 points with a 77% phase resolution with a 6/8^{ths} phase partial Fourier scheme, corresponding to the acquisition of 92 reconstructed to a matrix size of 160×120, and a typical spatial resolution $1.8 \times 1.8\,\mathrm{mm}$, with 25 cardiac phases.

4 Development and validation of lung water sequence

4.1 Motivation

In order to rapidly and robustly quantify the presence of dynamic changes in lung water, a constant low flip angle, multi-echo ultrashort echo-time (UTE) pulse sequence was developed, with a golden-angle radial-out sampling scheme and out-of-slice and fat saturation schemes, as shown in Fig. I.

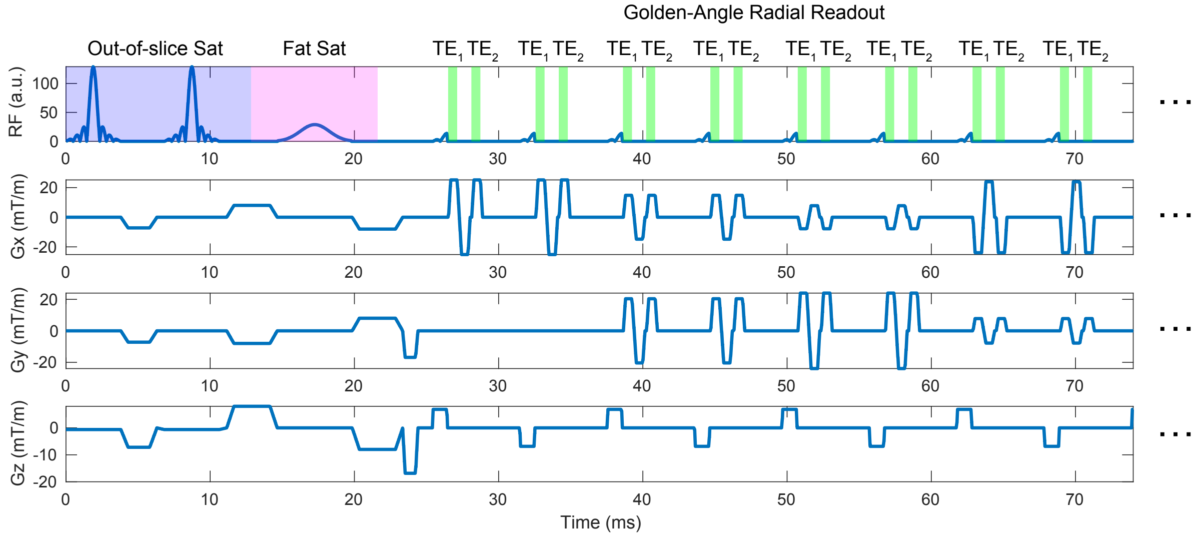

Figure I. Sequence diagram for the pulmonary proton imaging sequence, with the major parts of the acquisition detailed.

With the low flip angle used (5°) and the ability of UTE sequences to adequately resolve lung parenchyma, scan parameters were chosen to ensure an approximately linear response of signal intensity to proton density within the lung in its physiological range, under the assumption of constant T_1 (other scan parameters: TR $6\,\mathrm{ms}$; bandwidth $3\,\mathrm{kHz}$; TE 70 μs and $1.8\,\mathrm{ms}$ (chosen to be as close as SNR-optimal for a subsequent two-point T2* estimate whilst obeying sequence constraints, which is detailed later and follows on from previous work[56]); 512 readout points; $384\,\mathrm{mm}^2$ FOV; $10\,\mathrm{mm}$ thick; c.f. [57] for related details). Under the small flip angle regime, the effect of the RF pulse is approximately linear and the system enters the steady-state relatively quickly, leading to a benign variation with both B_1 and frequency, as illustrated via numerical Bloch equation simulation for the whole sequence (reading out the magnetisation after multiple echos) shown in Fig. II. Furthermore, if enough data is acquired this radial sampling scheme permits the post-hoc reconstruction of distinct periodic motion by the oversampling of the origin of k-space and the use of various binning algorithms.

For this application, we were explicitly mindful of the fact that exercise-induced nonperiodic motion may corrupt the acquisition and chose only a single signal phase and utilised a modified radial reconstruction algorithm to ensure that residual aperiodic motion artefacts would predominantly be aliased outside of the field of view of the lung. The TR of the saturation pulses was matched to the T_1 of subcutaneous fat at $3\,\mathrm{T}$ $(380\,\mathrm{ms})$, with each echo occurring at the minimum possible TR, taking into account hardware limitations and SAR concerns (i.e. TR $= 6\,\mathrm{ms}$). A total of $512 \times 12 = 6144$ radial spokes were obtained per echo, for a total scan duration of approximately 50-73 seconds, where the TR was minimised according to patient-specific PNS considerations.

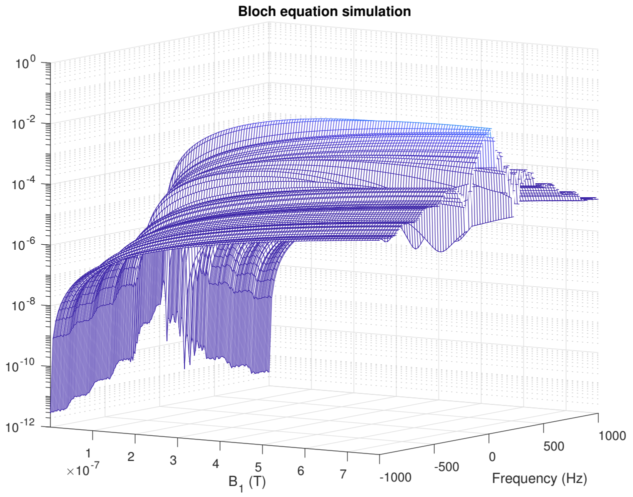

Figure II. The detectable proportion of magnetisation $|M_{xy}|$ at the first echo time as a function of frequency and hard pulse excitation power, determined via direct simulation of the Bloch equations. The frequency profile of the pulse is shown on the right hand side.

4.2 Image reconstruction

After acquisition, images were reconstructed as follows, using a modified version of the Berkley Advanced Reconstruction Toolkit (v0.6) recompiled locally to take advantage of Intel AVX512 linear algebra CPU extensions and running on a high-performance Linux workstation (dual Intel Xeon Gold 6252; 768 GiB RAM; Nvidia Titan V) to do numerical "heavy lifting" (NUFFT, parallel imaging reconstructions, etc);[5258] and Matlab.

Briefly, the reconstruction pipeline consisted of (1) constructing the appropriate total k-space sampling trajectory analytically regridding all acquired radial data through the non-uniform Fourier transform (NUFFT) and estimating central gradient delays by use of a gradient impulse-response function and explicit calculation and numerical optimisation. These were found to be effectively constant (and approximately isotropic, at 30 µs on all axes) for all acquisitions. The total number of radial spokes acquired is large, resulting in excellent k-space coverage within the radial envelope (c.f. Fig. III**a**). After this, whilst it is possible to reconstruct a naïve image at each echo directly without using compressed-sensing techniques, the resulting image obtained by simple DCF-corrected NUFFT is suboptimal compared to that obtained via a compressed-sensing reconstruction, is still subject to a few radial motion-artefact lines, and has a variable coil sensitivity map that would hinder quantitation and increase inter-patient variability (Fig. III**b**). To this end, (2) the delay-corrected acquired raw k-space data is collectively used in order to perform a multicoil sensitivity map estimation through the use of ESPiRIT (Fig. III**c**);[59] before (3) selecting a single cardiac phase sliding-window subset of radial spokes on the basis of a previously published quadratic self-binning algorithm,[60] implemented to ensure that motion artefacts remain inhomogeneous; and (4) reconstructing data via a parallel-imaging/compressed sensing method at twice the acquisition field of view with twice the desired number of points (and with a Kaiser-Bessel regridding parameter $\beta = 2$; ADMM $\rho = 0.005$; and multi-level Tikhonov regularisation $\lambda = 0.001$) to ensure that off-frequency-domain artefacts alias outside of the physiological field of view of interest (Fig. III**d**).[61] The resulting set of six same-phase images (Fig. III**e**) are then summed together (as complex images), exploiting the incoherence of any exercise-induced artefact present (which is expected to average to zero), and increasing SNR.

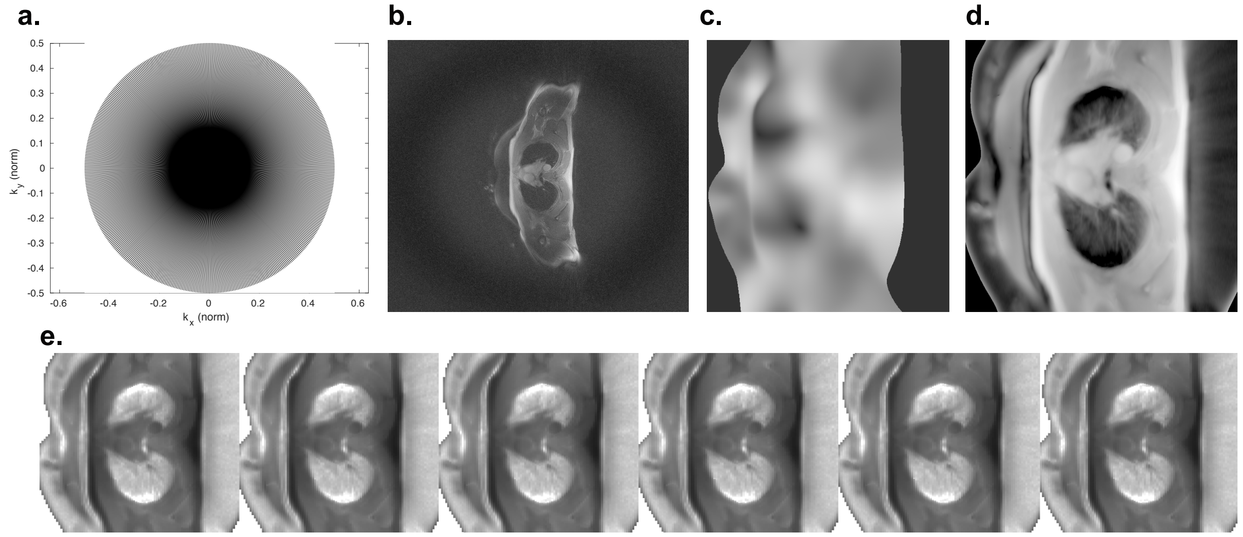

Figure III. A schematic of the reconstruction pathway. **a**: The overall acquired trajectory is densely oversampled and permits the super-Nyquist conventional reconstruction of an average image directly via simple NUFFT, shown in **b**. Despite an appropriate Density Compensation Function correction, this still demonstrates a significant central brightening artefact, which large results from motion artefacts being aliased as radial streaks. Following this, ESPiRIT based coil-compensation maps are created (shown in sum as (**c**) for the 32 channel array used) permitting the selection of a single phase and echo time, prior to its reconstruction via a parallel-imaging compressed sensing reconstruction algorithm in BART (**d**). This process can be repeated, selecting the same phase with different windows where exercise-induced noise is expected to be incoherent, producing a series of near-identical images (shown on a log-scale, **e**).

4.3 Point estimation

After the reconstruction of multi-echo images, a regularised, point-estimate of T_2^* was then computed from $T_2^* = \Delta\mathrm{TE}/\ln(S(\mathrm{TE}_1)/S(\mathrm{TE}_2))$; and image intensity scaled according to the gradient echo signal equation (Eq. (1)),

$$S = \rho_{^1\mathrm{H}} \frac{\sin\theta\left(1 - e^{-\mathrm{TR}/T_1}\right)}{1 - \cos\theta e^{-\mathrm{TR}/T_1}} e^{-\mathrm{TE}/T_2^*} \tag{1}$$

the acquisition TR, and an assumed constant literature value of $T_1 = 1\,\mathrm{s}$,[56] as it was not feasible to measure this in the UTE environment of the lung whilst the patient is exercising, within the punishing time constraints of the exercise protocol.

This does not represent a significant restriction, under these circumstances with a calibrated flip angle of 5º, Eq. (1) becomes equivalent to $S \approx \rho_{^1\mathrm{H}}\left(\theta + \mathcal{O}(\theta^3 \cdot f(\mathrm{TR}/T_1)\right) e^{-\mathrm{TE}/T_2^*}$ and thus the relative error due to T1 effects is small, as illustrated graphically in Fig. IV**a** which shows the variation of S as a function of T_1 and θ. In order to assess the relative error in this point estimate for T2*, consider again that the relative error for this estimate can be determined by the expression

$$\left|\frac{\delta T2^*/T2^*}{\delta S/S}\right| = \frac{\sqrt{e^{2\mathrm{TE}_1/T2^*} + e^{2\mathrm{TE}_2/T2^*}}}{(\mathrm{TE}_2 - \mathrm{TE}_1)/T2^*}, \tag{2}$$

and a desire to minimise this function arises, whilst still being subject to the bandwidth and gradient performance limitations of the scanner. An illustration of this error as a function of the second echo time and intrinsic true T_2 of the tissue is shown in Fig. IV**b**.

The resulting images were saved as 32-bit DICOMs, and manually masked by blinded observers to the lung field. The reconstruction process took approximately three hours per patient, limited predominantly by memory bandwidth and available computer cores. It is expected that the resulting data are approximately linear in proton density within the lung (i.e. where the two echoes acquired are sensitive to the T_2^* of the tissues).

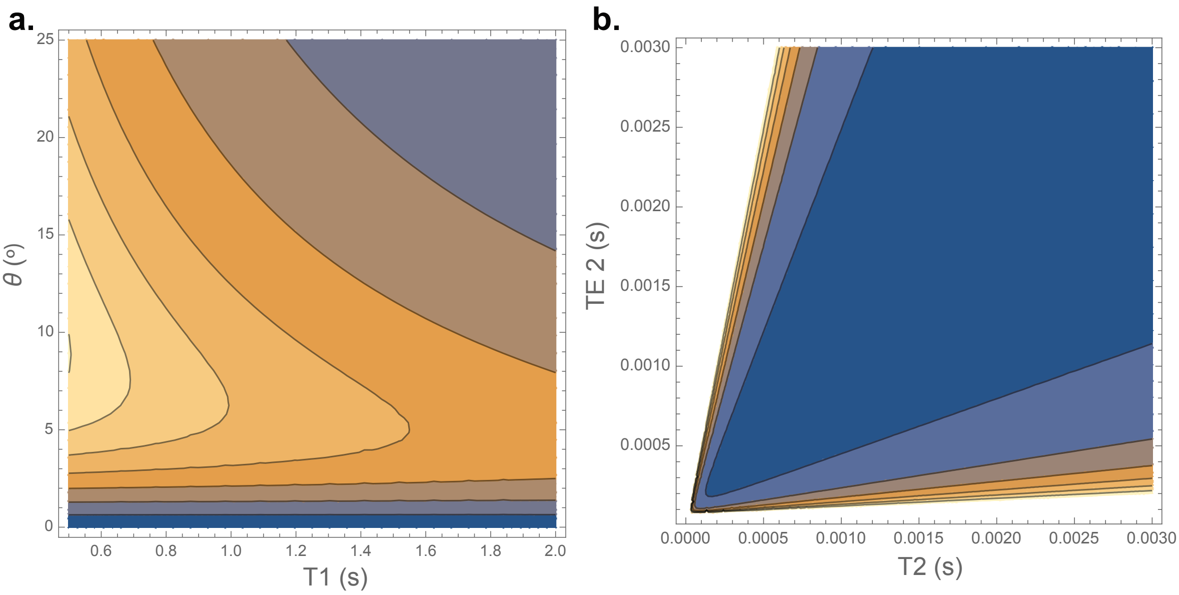

Figure IV. **a**: An illustration of the relative sensitivity of the derived MR-signal, plotted for the experimental parameters used as a function of T_1 and assuming a literature value of T_2 (as specified in Eq. (1)); and **b**: an illustration of the relative error specified in Eq. (2).

4.4 Phantom validation

This results in a compact sequence that is able to quantify a linear increase in proton density in challenging electrodynamic environments. We note that it provides an acquisition time compatible with the exercise duration of heart failure patients, and the ability to determine, *post hoc*, an appropriate number of rotational interleafs to resolve tissue with minimal motion-induced artefact – but it is also the case that there is substantial room for future development, for example, by the adoption of an appropriately artefact-proof 3D k-space trajectory. We verified the sequence within the $\sim 100\,\mu\mathrm{m}^3$ pores of a sponge phantom that was intended to act as a mimetic of the physiology of the lung parenchyma with a similar T_1 and T_2^*. A clearly linear increase in pulmonary proton (R1/R2 corrected UTE MR) signal with increasing water density was observed (Fig. V), as is expected.

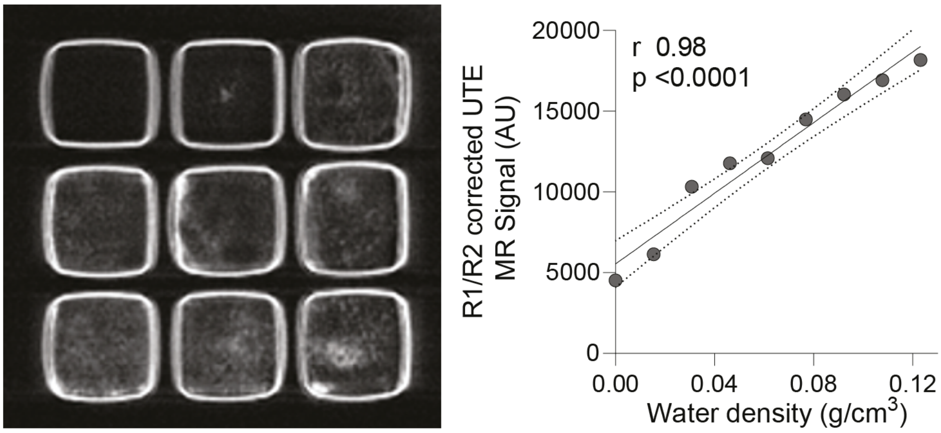

Figure V. Sponge phantom validation demonstrating a linear increase in determined proton signal with increasing water content predetermined to be set within the lower-end of the physiological range of hydration, where the quantification accuracy is expected *a priori* to be worse.

We note that similar MR approaches to accurately quantifying lung-water content have been extensively histologically and gravimetrically validated.[62] Accordingly, a linear look-up table was applied to lung images for visualization of pulmonary proton density signal (c.f. Fig. VI).

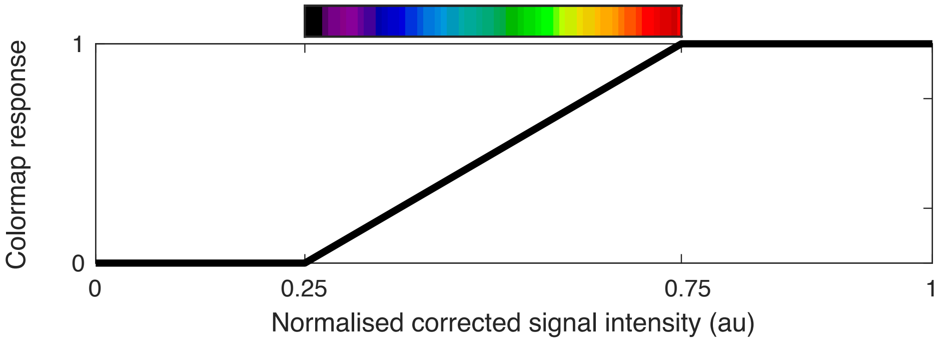

Figure VI. Look-up table for pulmonary proton signal images, based on the 'UCLA' colour scheme originally determined from the open-source DICOM viewer Weasis.

4.5 Test-retest repeatability at rest

Having developed this technique, a quantitative measure of its sensitivity to (likely small) pathological changes is required. To assess the test/re-test repeatability of the pulmonary proton density imaging at rest, healthy volunteers $(n = 11)$ were scanned on two separate occasions and blinded analysis performed by the same analyst: it is clearly expected that the same answer should be obtained on both occasions. As shown in the Bland-Altman plot Fig. VII, the test-retest repeatability of the measures was excellent, with a mean difference of $78\,\mathrm{a\,u}$; 95% CI -444, 601.

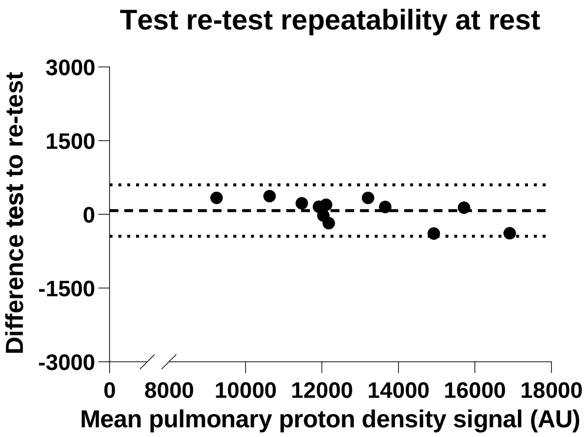

Figure VII. Bland Altman analysis to assess test re-test repeatability at rest.

4.6 Test-retest repeatability at stress

Similarly, to assess test re-test repeatability of the pulmonary proton density imaging under exercise-induced stress, healthy volunteers $(n = 11)$ were scanned following 6 minutes exercise on two separate occasions and blinded analysis performed by the same analyst. As shown in Fig. VIII, test-retest repeatability of the method was also excellent (mean difference -48, 95% CI -770, 674). We note that this is arguably as much a physiological measure of repeatability as well as a technical one.

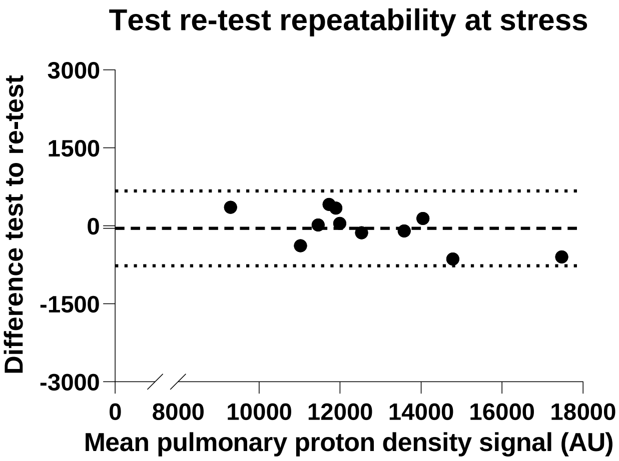

Figure VIII. Bland Altman analysis to assess test re-test repeatability at stress.

4.7 Intra-observer variability

Next, we wished to assess intra-observer reproducibility of the technique. Blinded analysis of the study participants was hence conducted on two separate occasions by the same expert analyst, contouring all patient data in the study. Bland Altman analyses confirmed good agreement, as shown in Fig. IX (mean difference 181; 95% CI -491, 879).

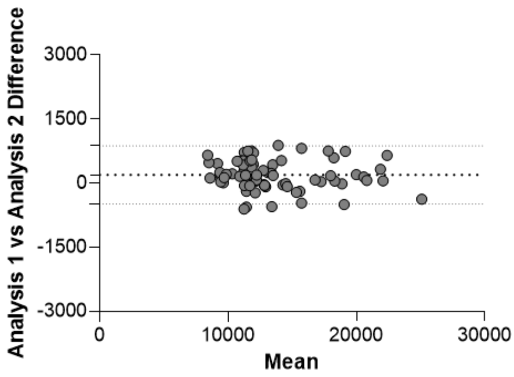

Figure IX. Bland Altman analysis to assess intra-observer variability.

4.8 Inter-observer variability

Finally, to assess inter-observer variability two cardiologists independently contoured pulmonary fields. These blinded inter-observer variability studies were performed for patients in the HFpEF group and again demonstrated good levels of agreement (c.f. Fig. X; mean difference -81; 95% CI -1000, 845).

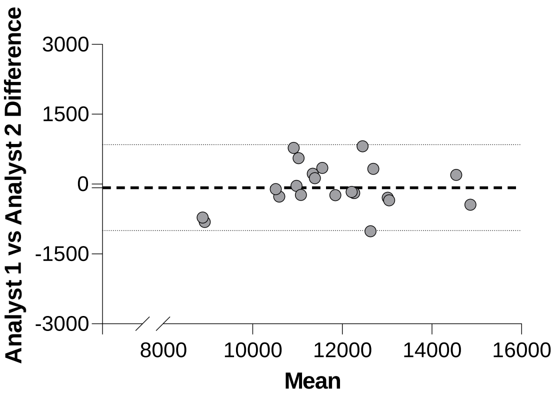

Figure X. Bland Altman analysis to assess inter-observer variability.

5 Results supplement: additional analyses

The inclusion of patients with cardiac amyloidosis in this study was prespecified in order to reflect the most severe end of the spectrum of diastolic dysfunction, and to provide a positive control (particularly for the lung water imaging). Different pathophysiological mechanisms exist across this spectrum and opinions as to whether cardiac amyloidosis is truly a form of HFpEF vary. Additional *post hoc* analyses of ordered medians excluding the amyloidosis group are therefore provided.

5.1 Supplemental Table I: Baseline characteristics with statistical comparisons excluding cardiac amyloidosis group

	**Controls n=11**	**T2DM n=9**	**HFpEF n=14**	Cardiac Amyloid n=9	**Group p***
Age (years)	64 [59, 68]	63 [59, 71]	69 [58, 76]	78 [72, 85]	0.10
Male (%)	7 (64%)	8 (89%)	8 (57%)	8 (89%)	0.99
BMI (kg/m^2)	24 [21, 25]	30 [26, 34]	31 [29, 38]	26 [24, 30]	<**0.001**
SBP (mmHg)	126 [124, 133]	138 [125, 156]	148 [123, 158]	119 [104, 139]	0.18
DBP (mmHg)	77 [67, 81]	79 [75, 83]	80 [72, 88]	69 [60, 75]	0.16
Resting HR (bpm)	68 [55, 76]	66 [64, 78]	70 [65, 86]	80 [77, 96]	0.11
Exercise HR (bpm)	98 [92, 105]	96 [83, 106]	96 [84, 118]	109 [93, 120]	0.71
Statin	1 (9%)	8 (89%)	8 (57%)	6 (67%)	
ACEi/ARB	-	5 (56%)	9 (64%)	3 (33%)	
Beta blocker	-	1 (11%)	9 (64%)	3 (33%)	
CCB	1 (9%)	1 (11%)	3 (21%)	0 (0%)	
MRA	-	0 (0%)	3 (21%)	2 (22%)	
Diuretic	-	1 (11%)	9 (64%)	9 (100%)	
Aspirin	-	0 (0%)	3 (21%)	2 (22%)	
Anti-coagulant	-	0 (0%)	7 (50%)	5 (56%)	
Oral hypoglycemic	-	8 (89%)	2 (14%)	1 (11%)	
Tafamidis	-	-	-	2 (22%)	
HFA-PEFF score	0 [0, 1]	2 [0, 2]	5 [4, 6]	6 [5.5, 6]	<**0.001**
Atrial fibrillation	0 (0%)	0 (0%)	7 (50%)	4 (44%)	**0.008**
NYHA class:					
**I**	11	8	2	0	
**II**	0	1	11	6	
**III**	0	0	1	3	
**IV**	0	0	0	0	
HbA1c (mmol/l)	5.5 [5.3, 5.7]	7.2 [6.9, 7.7]	5.1 [4.7, 5.6]	5.8 [5.5, 6.4]	0.71
NT-proBNP (pg/ml)	78 [49, 91]	38 [24, 80]	724 [245, 1606]	4217 [1964, 9978]	<**0.001**
E/e′ ratio	6.9 [5.4, 8.0]	6.8 [6.1, 8.0]	8.2 [7.2, 9.7]	19.4 [13.5, 21.6]	**0.02**
sPAP (mmHG)	9.1 [7.8, 10.8]	9.3 [8.6, 25.3]	14.6 [12.2, 35.4]	40.7 [22.5, 53.5]	**0.001**
Native T1 (ms)	1143 [1116, 1163]	1152 [1124, 1170]	1172 [1154, 1196]	1346 [1278, 1349]	**0.009**
PCr/ATP ratio	2.15 [2.09, 2.29]	1.71 [1.61, 1.91]	1.66 [1.44, 1.89]	1.30 [1.16, 1.53]	<**0.001**

Table I. Participants' baseline characteristics, *excluding cardiac amyloidosis*. Continuous variables shown are as median with interquartile range. Categorical variables are $n(\%)$. BMI indicates body mass index; SBP, systolic blood pressure; DBP, diastolic blood pressure; HR, heart rate; ACEi, angiotensin converting enzyme inhibitor; ARB, angiotensin receptor blocker; CCB, calcium channel blocker; MRA, mineralocorticoid receptor antagonist; NYHA, New York Heart Association; HbA1c, glycated haemoglobin; NT-proBNP, N-terminal pro-brain natriuretic peptide; sPAP, systolic pulmonary artery pressure; PCr, phosphocreatine; and ATP, adenosine triphosphate.*p values reported are the result of Jonckheere-Terpstra test for ordered medians across the groups or Fisher's exact test for categorical variables.

5.2 Supplemental Table II: Cardiac and pulmonary indices at rest and during 20 W exercise with statistical comparisons excluding cardiac amyloidosis

These are detailed in Table II, overleaf.

6 Supplemental analyses

6.1 Associations between resting heart rate, blood pressure, obesity and pre-diabetic states on outcomes

There were no significant correlations between heart rate (HR) and stress-inducible change in pulmonary proton density for resting HR ($r = 0.29$, $p = 0.06$), peak stress HR ($r = 0.08$, $p = 0.6$), change in HR ($r = -0.22$, $p = 0.15$), or blood pressure (systolic BP $r = -0.24$, $p = 0.13$, diastolic BP $r = -0.11$, $p = 0.49$). When controlling for the variables of age and blood pressure, the relationship between PCr/ATP and percentage change in lung water remained statistically significant ($p = 0.02$). There were no significant correlations between stress-inducible lung water change and BMI ($r = 0.09$, $p = 0.59$) or HbA1c ($r = 0.04$, $p = 0.82$).

	Controls (n=11)			T2DM (n=9)			HFpEF (n=14)			Cardiac Amyloid (n=9)			Group p†	
	**Rest**	**20 W**	**p***	**Rest**	**20 W**	**p***	**Rest**	**20 W**	**p***	Rest	20 W	p*	**Rest**	**20W**
LVEDV (ml)	157 [119, 165]	162 [124, 191]	**0.01**	172 [140, 173]	193 [164, 203]	**0.009**	167 [112, 241]	177 [110, 255]	**0.046**	145 [131, 175]	167 [139, 182]	**0.02**	0.24	0.45
LVESV (ml)	51 [47, 54]	39 [34, 60]	0.12	54 [52, 74]	63 [59, 79]	0.55	64 [41, 106]	73 [39, 100]	0.95	67 [51, 94]	74 [60, 99]	0.10	0.26	**0.04**
LVSV (ml)	97 [73, 105]	111 [86, 140]	**0.004**	101 [84, 119]	112 [95, 131]	0.07	100 [71, 138]	109 [72, 143]	0.06	82 [70, 91]	79 [71, 101]	0.28	0.71	0.58
LVEF (%)	66 [61, 70]	73 [69, 76]	**<0.001**	62 [57, 68]	64 [61, 69]	0.44	61 [53, 69]	63 [52, 71]	0.38	55 [47, 60]	52 [45, 61]	0.44	0.11	**0.005**
LAV (ml)	92 [75, 112]	89 [75, 106]	**0.02**	81 [52, 96]	82 [63, 108]	**0.01**	82 [74, 94]	97 [82, 103]	**0.002**	100 [87, 149]	108 [102, 158]	**0.009**	0.42	0.84
EDV/s	3.5 [3.1, 4.5]	5.0 [4.4, 5.8]	**<0.001**	2.9 [2.5, 3.5]	4.0 [2.6, 4.3]	0.16	3.4 [2.3, 4.1]	3.2 [2.6, 3.8]	0.58	3.1 [2.3, 3.5]	2.6 [2.4, 3.2]	0.30	0.09	**0.006**
LV mass (g)	113 [81, 129]			135 [119, 143]			129 [94, 190]			200 [151, 255]			0.17	
LVMi (g/m^2)	57 [49, 64]			58 [52, 70]			59 [47, 87]			104 [81, 122]			0.70	
RVEDV (ml)	170 [118, 189]	183 [112, 189]	0.11	165 [141, 180]	182 [153, 210]	**0.009**	150 [107, 209]	177 [105, 227]	**0.01**	160 [107, 178]	174 [124, 187]	0.07	0.91	0.71
RVESV (ml)	61 [37, 90]	58 [31, 75]	**0.02**	64 [53, 74]	60 [53, 82]	0.48	58 [41, 86]	57 [43, 91]	0.50	66 [38, 96]	73 [45, 89]	0.09	0.86	0.75
RVSV (ml)	99 [74, 101]	111 [82, 134]	**0.008**	101 [81, 111]	122 [97, 126]	**0.009**	86 [68, 120]	101 [70, 137]	**0.006**	82 [66, 95]	87 [75, 104]	0.17	0.96	0.78
RVEF (%)	59 [53, 68]	69 [62, 71]	**0.004**	59 [57, 68]	67 [59, 69]	0.10	60 [54, 66]	65 [55, 70]	**0.04**	57 [47, 63]	58 [49, 63]	0.89	0.90	0.27
RAV (ml)	121 [85, 162]	116 [81, 149]	**0.004**	118 [80, 135]	126 [88, 141]	**0.04**	129 [98, 147]	143 [116, 154]	**0.002**	171 [141, 218]	203 [174, 244]	**0.009**	0.88	0.21
RV SV/ESV	1.4 [1.1, 2.2]	2.2 [1.6, 2.5]	**<0.001**	1.5 [1.3, 2.1]	2.0 [1.4, 2.2]	0.13	1.5 [1.1, 1.9]	1.8 [1.2, 2.3]	**0.03**	1.3 [0.9, 1.7]	1.4 [1.0, 1.7]	0.82	0.97	0.27
PDM (AU)	12030 [11599, 13678]	12014 [11091, 13960]	0.89	11535 [9690, 13034]	11656 [9492, 13007]	0.82	11588 [10753, 12353]	11845 [10995, 13137]	**0.002**	12597 [10874, 20242]	13163 [11512, 20984]	**0.004**	0.29	0.64
Δ PDM (AU)		-8 [-232, 282]			93 [-200, 179]			458 [72, 735]			981 [461, 1160]			**0.005**
Δ PDM (%)		-0.1 [-1.9, 2.1]			0.8 [-1.7, 1.9]			4.4 [0.5, 6.4]			6.4 [3.3, 10.0]			**0.009**

Table II. Cardiac and pulmonary indices on cardiovascular magnetic resonance imaging at rest and during 20W exercise stress *excluding cardiac amyloidosis*. Continuous variables shown are median [interquartile range]. LVEDV indicates left ventricular end-diastolic volume; LVESV, left ventricular end-systolic volume; LVSV, left ventricular stroke volume; LVEF, left ventricular ejection fraction; LAV, left atrial volume; EDV/s, end-diastolic volume per second; RVEDV, right ventricular end-diastolic volume; RVESV, right ventricular end-systolic volume; RVSV, right ventricular stroke volume; RVEF, right ventricular ejection fraction; RAV, right atrial volume; RV SV/ESV, right ventricular stroke volume to end-systolic volume ratio; PDM, pulmonary proton density mapping (lung water). *p values reported are results from Wilcoxon signed-rank test (rest vs 20 W within groups) or †Jonckheere-Terpstra test for ordered medians across the groups at rest and 20 W exercise.
